# Plasticity via feedback reduces the cost of developmental instability

**DOI:** 10.1002/evl3.202

**Published:** 2020-11-19

**Authors:** Remi Matthey‐Doret, Jeremy A. Draghi, Michael C. Whitlock

**Affiliations:** ^1^ Institute of Ecology and Evolution Universität Bern Bern 3012 Switzerland; ^2^ Department of Zoology and Biodiversity Research Centre University of British Columbia Vancouver BC V6T 1Z4 Canada; ^3^ Department of Biological Sciences Virginia Tech Blacksburg Virginia 24061

**Keywords:** Development, evo‐devo, phenotypic plasticity, simulation

## Abstract

Costs of plasticity are thought to have important physiological and evolutionary consequences. A commonly predicted cost to plasticity is that plastic genotypes are likely to suffer from developmental instability. Adaptive plasticity requires that the developing organism can in some way sense what environment it is in or how well it is performing in that environment. These two information pathways—an “environmental signal” or a “performance signal” that indicates how well a developing phenotype matches the optimum in the current environment—can differ in their consequences for the organism and its evolution. Here, we consider how developmental instability might emerge as a side‐effect of these two distinct mechanisms. Because a performance cue allows a regulatory feedback loop connecting a trait to a feedback signal, we hypothesized that plastic genotypes using a performance signal would be more developmentally robust compared to those using a purely environmental signal. Using a numerical model of a network of gene interactions, we show that plasticity comes at a cost of developmental instability when the plastic response is mediated via an environmental signal, but not when it is mediated via a performance signal. We also show that a performance signal mechanism can evolve even in a constant environment, leading to genotypes preadapted for plasticity to novel environments even in populations without a history of environmental heterogeneity.

**Impact Summary**

Phenotypic plasticity is the widespread property of living organisms of changing their morphology, physiology, or behavior in response to their environment. It is at the core of how organisms learn, interact with their environment, and how they might adapt to their environment during their lifetime. Although we have a good understanding of the selection pressures that can promote the evolution of plasticity, we have little understanding of the developmental mechanisms of plasticity and how different mechanisms affect the evolution of plasticity. In this article, we investigate two mechanisms by which a plastic response can be implemented; by sensing the state of the environment via an environmental signal or by sensing how well the organism is performing in a specific environment via a performance signal. We performed computer simulations of an evolving population by simulating the development of each individual with a network of gene interaction. We show that these different mechanisms for plasticity lead to different developmental consequences. More specifically, we show that the development tends to be more stochastic when sensing an environmental signal than when sensing a performance signal. The reason is that the performance signal acts as a negative feedback loop correcting undesired stochasticity in the development. The idea that a plastic genotype is more noisy than a nonplastic genotype is a commonly mentioned constraint to evolving plasticity. We show that this constraint depends upon the developmental mechanism for plasticity. Considering such a mechanistic model of developmental plasticity also allowed us to identify a way by which plasticity could evolve, not as a direct response to selection to plasticity but as a correlated side effect of selection for developmental robustness. Under such a scenario, we show that plasticity could evolve even in a constant environment.

Plasticity is the ability of a genotype to produce different phenotypes in different environments. Plasticity is a heritable trait (Côté et al., 2007; Landry et al., 2006; Li et al., 2006; Suzuki & Nijhout, 2006) that can have many ecological consequences (Forsman et al., 2008; Wennersten & Forsman, 2012): plasticity can increase population growth rate (Hughes et al., 2008; Wennersten & Forsman, 2012), broaden ecological niches (Smith & Skulason, 1996), reduce intraspecific competition (Forsman et al., 2008), and increase invasiveness (Sultan, 2000; Bock et al., 2018). Plasticity also affects population differentiation and the rate of speciation (Gray & McKinnon, 2007; Pfennig et al., 2010). Finally, plasticity can affect adaptive evolution (Price et al., 2003), including local adaptation (Arendt, 2015), responses to climate change (Charmantier et al., 2008; Nicotra et al., 2010; Hoffmann & Sgrò, 2011), and adaptation via genetic accommodation (Bock et al., 2018; Pigliucci et al., 2006). When a trait is adaptively plastic, the plasticity causes the organism to better match its environmental optimum.

By allowing organisms to sustain a high fitness in a range of environments, adaptive phenotypic plasticity would appear to provide a solution to any eco‐evolutionary problem. However, plasticity is not ubiquitous, which leads many authors to ask what limits its evolution (Agrawal, 2001; DeWitt et al., 1998; Moran, 1992; Murren et al., 2015; Scheiner et al., 1991; Scheiner & Holt, 2012; Swanson & Snell‐Rood, 2014; Van Tienderen, 1991; Tonsor et al., 2013; Van Kleunen & Fischer, 2005). There are a number of possible costs and limits to the evolution of plasticity (DeWitt et al., 1998; Snell‐Rood et al., 2010), but current empirical research on the costs of plasticity has been mainly inconclusive; costs have been rarely found and when found they are rather mild (Snell‐Rood, 2012; Snell‐Rood et al., 2010; Van Buskirk & Steiner, 2009; Van Kleunen & Fischer, 2005). One of the most commonly discussed costs is the idea that plasticity generates developmental noise and therefore great phenotypic variation, reducing fitness in comparison to more precise, constitutive expression of a phenotype (DeWitt, 1998; Scheiner et al., 1991; Tonsor et al., 2013).

Developmental noise, as defined in this article, refers to the phenotypic variation among clones of a specific genotype developed in the same environment. Developmental robustness is defined as the inverse of developmental noise. Developmental instability is heritable (McAdams & Arkin, 1997; Klingenberg & Nijhout, 1999) and is involved in various processes such as cellular specialization in multicellular organisms (reviewed in Losick & Desplan, 2008) and the penetrance of alleles (reviewed in Chalancon et al., 2012). Also, developmentally noisy traits experience reduced heritability (Tonsor et al., 2013) and hence reduced responses to selection. Importantly, developmental noise creates deviations from the targeted phenotype, which reduces fitness for a well‐adapted phenotype under stabilizing selection (Gavrilets & Hastings, 1994). However, developmental instability can also be beneficial as a form of bet‐hedging (Simons & Johnston, 1997; Seger & Brockmann, 1987; Veening et al., 2008). Evolution of developmental noise can affect the overall rate of adaptation (Wang & Zhang, 2011), as well as networks of gene interactions (Chalancon et al., 2012). Understanding the evolution of plasticity and developmental noise can therefore influence our thinking about many aspects of evolution and ecology.

In our simulation model, developmental noise arises from stochasticity in gene expression, translation, mRNA degradation, and protein degradation, which produce substantial variability among unicellular organisms (Chalancon et al., 2012). Noise can also propagate through regulatory interactions among genes (Pedraza & Van Oudenaarden, 2005), and this effect increases with the extent of the regulatory cascade (Hooshangi et al., 2005). Multicellular organisms may have different and additional sources of random phenotypic variability, motivating us to treat expression noise as representative of more general sources of phenotypic noise.

### COSTS AND LIMITS ON THE EVOLUTION OF PLASTICITY

The idea that plasticity comes at a cost of developmental instability is one of the most popular constraints discussed in the literature (e.g., Debat & David, 2001; DeWitt, 1998; Scheiner et al., 1991; Snell‐Rood et al., 2010; Tonsor et al., 2013; Wilson & Yoshimura, 1994; Yoshimura & Shields, 1995). Because a plastic response might require a complex regulatory mechanism and because a more complex regulatory cascade is expected to be more stochastic, it follows that plastic traits are likely to be more developmentally noisy than their nonplastic counterparts (see also DeWitt, 1998).

The prediction of the relationship between plasticity and developmental robustness is based on an intuitive prediction that does not distinguish among the diverse developmental pathways by which a plastic response can be implemented (as criticized by Snell‐Rood et al., 2010). The simplest developmental mechanism for a plastic response is one where individuals sense an environmental signal and respond to it. *Daphnia* can respond to kairomones released by predators such as fish, backswimmers, or midge larvae, and they also respond to chemicals released by macerated conspecifics (Laforsch et al., 2006). In the presence of such signals, several species of *Daphnia* develop a long helmet on their head that protect them against these predators. Other examples of plastic responses involving an environmental signal include the Pennsylvanian meadow vole's coat thickness, which in the offspring is dependent on the duration of daylight experienced by the mother (Lee & Zucker, 1988) and the East African *Acridoid* Grasshoppers whose melanin deposition for camouflage is greater after a fire (Rowell, 1972).

An alternative mechanism is what Snell‐Rood (2012) calls “developmental selection” (also known as “somatic selection” or “epigenetic selection”; Sachs, 1988; West‐Eberhard, 2003). Developmental selection has two components: First, the genotype must produce a range of phenotypes, and second, the genotype must assess the performance of each phenotype and bias subsequent development toward the highest performing phenotypes (Snell‐Rood, 2012). The essential distinction between simple plasticity and developmental selection is that the latter involves a performance signal that integrates information from both development and the environment and act like a negative feedback loop (see also Bhalla & Iyengar, 1999; Becskei & Serrano, 2000). We will therefore contrast plasticity in response to an environmental signal with plasticity in response to a performance signal.

Although a performance signal as a mechanism for plastic responses has been largely ignored in the evolutionary literature, the reality is that such mechanisms are very common in nature (Snell‐Rood, 2012). In humans, bones respond plastically to impact loading by increasing their sizes, mineral content, and density (reviewed in Zernicke et al., 2006). In tennis players, for example, bone mineral content is 13% higher on the dominant arm than that on the nondominant arm (see Ducher et al., 2004 and Sanchis‐Moysi et al., 2011 for similar observations in tennis players on gluteal muscles). Osteocytes serve a key role in sensing the resistance of the bone when bearing mechanical stress and communicate this information to osteoclasts and osteoblasts that will, respectively, degrade and synthesize bone tissue (Zernicke et al., 2006). Other tissues show similar plastic response to performance signals such as cell walls in plants (Tiré et al., 1994) and muscles in animals (Hoppeler et al., 2011). Other examples include any trial‐and‐error learning behaviors in, say, foraging, or for learning associations between cues and rewards (Dukas, 2008; Papaj & Prokopy, 1989), vertebrates’ adaptive immune system (self‐ vs. nonself‐distinction; Litman et al., 1993; Nemazee, 2006), and neuroplasticity in the brain (Luo & O'Leary, 2005; Song & Abbott, 2001). More examples and evidence of the commonality of performance signal mechanisms are reviewed in Snell‐Rood (2012).

### HYPOTHESES

Plastic developmental pathways tend to be more complex than nonplastic ones, and more complex developmental pathways tend to be more noisy (Hooshangi et al., 2005). Also, plasticity requires sensitivity to the environment, which allows noise in the signal to affect the phenotype. At the same time, feedback loops have been shown to buffer against developmental noise, hence increasing developmental robustness (Becskei & Serrano, 2000; Bhalla & Iyengar, 1999). A performance signal mechanism involves a negative feedback loop, and therefore we hypothesize that plasticity in response to an environmental signal would come at a cost of being developmentally less robust, but this cost would be attenuated or even reversed when the organism evolves plasticity via performance signal.

Because performance signals might increase developmental robustness (Becskei & Serrano, 2000; Bhalla & Iyengar, 1999), one can expect that a performance signal mechanism could evolve even in a constant environment, to increase developmental robustness. Several authors have suggested that organisms using a performance signal are more likely to respond adaptively to a novel environment (Frank, 2011; Hull et al., 2001; Snell‐Rood, 2012), and this hypothesis has been discussed thoroughly in Snell‐Rood et al. (2018). Hence, we also hypothesize that performance signal use can evolve in a constant environment to increase developmental robustness and create preadaptation through a plastic response to novel environments: organisms that have never encountered any environmental heterogeneity might nonetheless be adaptively plastic.

We investigate these two hypotheses through numerical simulations with a numerical model called ENTWINE (Draghi & Whitlock, 2015), which we modify here. Our goal is to simulate the evolution of network of genes that are regulated by other genes’ transcription factors. Parameters of the model are inspired from the empirical literature as we strive to model gene network interactions with as much realism as can be computationally tractable.

## Methods

We simulated the evolution of a developmental network through computer simulations, using a modified version of the program ENTWINE (Draghi and Whitlock 2015). For each individual, ENTWINE simulates the development of a phenotype in a single‐cell organism, based on a small interacting network of genes and their products. ENTWINE uses a modified Gillespie algorithm (Gillespie, 2007) to model stochastic reactions of mRNAs and proteins. This framework sets a time step *τ* that aims at balancing the computational time and controlling the error produced by the discrete‐time approximation. Each individual cell develops for a constant period of 300 min. ENTWINE also allows populations of such individuals to evolve over time, with stabilizing selection on the organism's phenotypic value at the end of development. For computational convenience, the evolutionary algorithm uses a panmictic populations of haploid individuals using a Wright‐Fisher model. Plasticity is specific to particular phenotypic traits and environments (Wagner, 2005). In this article, we will be focusing on a single phenotypic trait and a specified set of environmental conditions, allowing us to talk about “plastic and nonplastic genotypes” without ambiguity.

For a detailed description of the model's methods and sources for parameter and design decisions, see Draghi and Whitlock (2015). Below, we sketch out the essential features and provide full details about the additions to the model that are specific to this article.

### DEVELOPMENT OF AN INDIVIDUAL

The phenotype of an individual is determined by simulating its development using a model of a network of gene interactions. We modeled three types of genes: *regulatory genes*, *phenotype genes*, and *signal‐binding genes*. The *regulatory genes* code for transcription factors that can only regulate the transcription rates of themselves or of other genes. The *phenotype genes*, such as *regulatory genes*, can regulate other genes but can also directly affect the growth of the measured phenotypic trait. The *signal‐binding genes* produce a protein that can interact with an environmental signal through cooperative binding, described in the subsection “signals” below. The protein complex formed by this interaction between the protein and the environmental signal may then regulate the expression of other genes in the same way as a transcription factor. None of these types of gene can mutate to any other type, but genes may mutate in several ways, including deletion and duplication of an entire gene. In these simulations, a gene needs an enhancer to be expressed; therefore, nothing can be expressed in the system unless there are some transcription factors present by default. As a consequence, we provide a “basic transcription factor” that is inputted into the system at a constant rate and can eventually start the expression of one or more genes in the system. This basic transcription factor does not evolve in these simulations.

In ENTWINE, each gene is made of two parts: the *cis*‐regulatory region and the coding region. The *cis*‐regulatory region is made of a number of distinct binding sites, set to 20 for the simulations reported here. Each binding site is associated with a *cis*‐regulatory effect and a binding affinity (an integer implicitly representing the number of amino acid mismatch between the protein's binding domain and the binding site). The coding region for transcription factors contains two types of information: the protein's inherent regulatory effect (a positive or negative value) and which one of the possible *cis*‐regulatory binding motifs (of the 20 allowed possibilities) it matches, and therefore binds to. By implementing a model of regulation grounded in biophysical mechanisms, we allow genes to respond to external and internal signals and affect other genes’ expression by *cis*‐regulatory interactions. The rate of transcription is modeled with a Michaelis‐Menten‐like equation representing the binding and action of activators and repressors. The model tracks the discrete number of transcription factors in which changes are stochastic, affected by transcription, and decay events. To focus on *cis*‐regulation, we fix the rate of translation and the rates of decay of both mRNA and proteins, for all genes. Therefore, mutations only affect regulation by altering the rate of transcription of a gene.

We measured a single phenotypic trait on which selection is applied. The *phenotype genes* produce a protein that catalyzes growth of the target phenotype. The phenotype starts at zero and never decays, hence simulating a case of irreversible plasticity (Foster et al., 2015). The phenotype increases at a rate that is proportional to the number of copies of the phenotypic catalysis proteins multiplied by their specific phenotypic effects.

Many aspects of the developmental system are subject to mutation in each generation. There are six types of mutations: duplications, deletions, and four types of mutations that can influence the effect of a given protein on gene expression. Mutations on the effect of the protein can affect (1) the protein's inherent regulatory effect, (2) the binding affinity between the protein and the binding site of the target gene, (3) the binding site targeted by the protein, or (4) the *cis*‐regulatory effect of the binding site. More information on the mutation rates and distribution of effect sizes for each of these mutation types is in the Supporting Information of Draghi and Whitlock (2015). Duplications happens at a rate of 10^−8^ per replication per genome and deletions happen at a rate 10^−7^ per replication per gene.

### SIGNALS

The signal‐binding genes react to an endogenous signal produced in response to some information available to the organism. We assume that this information is provided by structures outside the focal evolving gene network. As a result of this information capture, a signal protein is input to the system at a rate proportional to the strength of the signal being measured. For the environment signal, the rate of input is $P_{e}$, where $P_{e}$ is the optimal phenotype in the environment e. For the performance signal, the rate of input is |$P_{t}-P_{e}$|, where $P_{t}\;$is the current phenotype at time t during development. In both cases, in the system, the signal decays at the same rate as do proteins and is therefore subject to the same stochasticity in its decay. The environmental signal mechanism was present in the previous version of ENTWINE (Draghi and Whitlock 2015); the performance signal was added to the program for this project.

Once in the system, the signal can affect gene expression through cooperative binding with the protein product of a *signal‐binding gene*, creating a heterodimer that acts as a transcription factor. Let *S* be the signal's concentration. Cooperative binding is modeled with a Hill equation (Hill, 1910) $S_{e}=\frac{S^{n}}{K^{n}+S^{n}}\;$, where n is the Hill coefficient, K is the dissociation constant, and $S_{e}$ is the effective signal, the concentration available to act as a transcription factor. The Hill coefficient n is specific to each gene and takes a random value uniformly distributed between 1.0 and 5.0 when genotypes are constructed at the start of the simulation (see section STARTING CONDITIONS below). Cooperative binding is newly added to the code for ENTWINE for this article.

### EVOLUTION

We simulated the evolution of this network of gene interaction over time. Individuals in ENTWINE are haploid, hermaphroditic, and randomly mating. Evolution occurred with nonoverlapping generations with a constant population size of 10,000 individuals. The target phenotype was subjected to Gaussian stabilizing selection, based on the phenotype of the individual at the end of development. If, at the end of its development, an individual phenotype is *P* in an environment *e* where the optimal phenotype is *P_e_*, then the fitness of this individual is $\exp[-\frac{{\left(P-P_{e}\right)}^{2}}{\omega }]$, where $\omega =5\;\times \;10^{-5}\;$represents the strength of selection.

The code along with an example of an input file with all parameters is available at https://github.com/RemiMattheyDoret/ENTWINE.

### INPUT PARAMETERS AND TREATMENTS

We explored two types of environmental patterns: a constant environment and a spatially heterogeneous environment. The constant environment treatment is to study evolution of plasticity as a correlated side effect of the evolution of developmental robustness. The spatial heterogeneity treatment is to study whether plasticity comes at a cost of developmental instability and how different signals can affect this cost.

In each type of environment, we considered three treatments: *Environmental Signal*, in which there is an environmental signal that the organism can potentially evolve to sense; *Performance Signal*, in which there is a performance signal that the organism can potentially evolve to sense; and finally, *No Signal*, in which no cue is present (and therefore no way to evolve plasticity). This 2 × 3 design yields a total of six treatments, and we performed 200 replicates simulations for each treatment.

**Figure 1:**
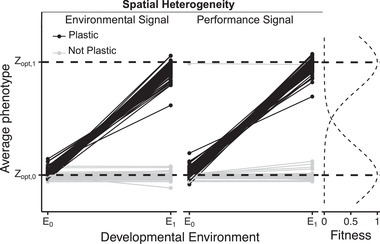
Reaction norms for the *Environmental Signal* and *Performance Signal* treatments under spatial heterogeneity. Each line represents a single genotype (a single simulation) and links the average phenotypes in both environments. Reaction norms that are steep enough to be considered plastic (see *Methods*) are represented in black; nonplastic genotypes are represented in gray. The horizontal dashed lines represent the optimal phenotypes in each environment. On the right panel are the fitness functions in both environments.

We followed the evolution of each population for at least 100,000 generations. Under spatial heterogeneity, half of the simulations of each of the three treatments were randomly chosen and were extended to 200,000 generations. Gaussian stabilizing selection is applied on a single phenotypic trait. We consider two environments, a *low* and a *high environment* in which the optimal phenotypes are 1000 and 3000, respectively (the fitness functions are displayed on the right side of Fig. 1). For the constant environment treatment, individuals only experience the *low environment*. With spatial heterogeneity, each individual is randomly put in either environment in each generation (resulting in a migration rate of 0.5 among environments). This extreme scenario was chosen to have a high incentive for evolving a plastic response. Because our goal is to establish a proof of concept and not to estimate how different input parameters will impact the observed statistics and, also because simulations are computationally very expensive (a single simulation can take up to a month to run), we did not further vary the parameters such as the strength of selection or the optimal phenotypes to reach in the two environments.

Note that because the protein production is essentially a Poisson process and because the variance of a Poisson distribution equals its mean, a genotype that produces more protein products ought to be more noisy. Per consequence, genotypes that produce a large phenotype would tend to be more noisy too, hence causing a preference for targeting the low environment rather than the high environment for nonplastic genotypes.

### STARTING CONDITIONS

Each simulation started with a population of cloned haploid individuals with a genome containing three *regulatory* genes and three *phenotype* genes. In the *Environmental Signal* treatment, organisms start with an extra three environmental signal genes and in the *Performance Signal* treatment, organisms start with an extra three performance genes. Because gene duplication and gene deletion are possible, the number of genes in a representative genotype after 100,000 generations of evolution generally differed from the starting number of genes.

Every simulation started with a population of clones founded by a unique ancestor. At the beginning of every simulation, before simulating the first generation, potential ancestral genotypes were randomly produced until a genotype that has a mean fitness between 0.15 and 0.25 is found. This fitness assay is measured in the *low environment*. This genotype is then cloned to create one starting population.

### MEASURING PLASTICITY AND ROBUSTNESS

We sampled the most common genotype in the population at the last generation to measure the statistics of interest. For the treatments *Environmental Signal* and *Performance Signal*, under spatial heterogeneity, we also sampled other generations during the run to investigate the evolutionary dynamics. Plasticity and developmental robustness were then measured for each sampled genotype.

To measure plasticity, we redeveloped each genotype 50,000 times in the *low environment* and 50,000 times in the *high environm*ent. Because the vast majority of genotypes were either very plastic or not plastic at all (Fig. 1), instead of considering plasticity as a quantitative trait, we categorized genotypes as plastic based on whether the average phenotypes between the two environments was greater than Δ*P* = 400 (i.e., greater than 20% of the difference in the optima). We have explored different threshold values for Δ*P* (100, 200, and 800), and the specific threshold did not affect the general conclusions (data not shown).

To determine the developmental robustness of a genotype, we used the 50,000 redevelopments of this genotype described above in the *low environment*. We define the developmental noise as the standard deviation in the realized phenotypes.

## Results

Figure 1 shows the reaction norms for *Performance Signal* and *Environmental Signal* under spatial heterogeneity at the end of the simulations. In these treatments and with very few exceptions, genotypes are either very adaptively plastic or not plastic at all. With a single exception, the nonplastic genotypes target the lower environment (the environment in which the initial fitness was required to be between 0.15 and 0.25).

### PLASTICITY AND DEVELOPMENTAL ROBUSTNESS

**Figure 2:**
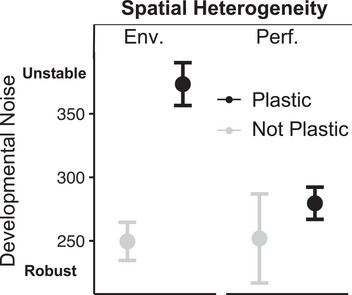
Average developmental noise for the *Environmental Signal* and *Performance Signal* treatments under spatial heterogeneity, separating the plastic (in black) from the nonplastic genotypes (in gray). Developmental robustness was measured in the low environment. See Figure S1 for developmental noise per individual simulation for all treatments. Error bars are standard errors. See Figure S2 for the evolution of developmental noise over time for these two treatments. Developmental noise is defined as the standard deviation of the phenotypes created by replicate development of identical genotypes.

Because the performance signal involved a negative feedback loops, and because such loops have been shown to buffer against developmental noise (Becskei & Serrano, 2000; Bhalla & Iyengar, 1999), we hypothesized that plasticity would come at a cost of increased instability when implemented via an environmental signal but that this cost would not exist (or even be reversed) when implemented via a performance signal. Figure 2 shows that nonplastic genotypes are less developmentally noisy than plastic genotypes in the *Environment Signal* treatment (Welch *t*‐test: 79 < δ < 168, *P* < 5 × 10^–7^), but not in the *Performance Signal* treatment (Welch *t*‐test: –53 < δ < 108, *P* ∼ 0.47). Importantly, plastic genotypes using the performance signal are less developmentally noisy than plastic genotypes using the environmental signal (Welch *t*‐test: 52 < δ < 136, *P* < 3 × 10^−5^). We observe a significant interaction between the signal and whether a genotype is plastic after controlling for the main effects of signal and whether a genotype is plastic (OLS regression: *P* < 0.05).

In the *Environmental Signal* treatments, nonplastic genotypes are significantly more robust than plastic genotypes; however, we observe substantial variance in developmental robustness within treatments and an important overlap among individual values among treatment (Fig. S1). Results for other treatments are also present in Figure S1.

Developmentally robust genotypes may face constraints preventing the evolution of plasticity (Draghi, 2019). In Figure S2, we see that the genotypes that eventually evolve plasticity have lower robustness than those that do not evolve plasticity. This difference among plastic and nonplastic lineages persists during the entire simulation in the *Environmental Signal* treatment. Therefore, the cost of plasticity (lower fitness caused by extra developmental noise) persists with *Environmental Signal* throughout the duration of the simulations. However, with the *Performance Signal*, the initial difference in robustness is eliminated by the end of the simulations.

### PERFORMANCE SIGNAL MAKES IT EASIER TO EVOLVE PLASTICITY

We hypothesized that a genotype may evolve to use performance signal even in a constant environment and, as a result, be adaptively plastic to environments that have never been encountered in the evolutionary history of the lineage. This prediction results from the idea that a performance signal can increase developmental robustness and hence can be beneficial even in a constant environment, therefore making the organism plastic as a side effect. However, some genotypes in a constant environment with an environmental signal evolved some level of plasticity, which was unexpected. Therefore, we wanted to investigate whether plasticity in constant environments evolves more often in the *Performance Signal* treatment than in the *Environmental Signal* treatment.

Comparing the fractions of simulations that evolved a plastic response in both treatments is made a little more complicated because some of the starting genotypes were already plastic. In our model, an organism needs to use some cue to initiate the development. For the majority of genotypes, this cue is the “basic transcription factor” (see *methods*), but some genotypes used either the performance signal or the environmental signal to initiate their development. In fact, with the constant environment, 30% and 37.5% of genotypes started out plastic for the *environmental signal* and *performance signal* treatments, respectively. Most starting plastic genotypes lost their plasticity very early on, with only 10% and 9.3% of the genotypes that started plastic remaining plastic during the whole simulation for the *environmental signal* and *performance signal* treatments, respectively. Of course, some of these lineages that have lost their initial plastic behavior regained it during the run.

**Figure 3:**
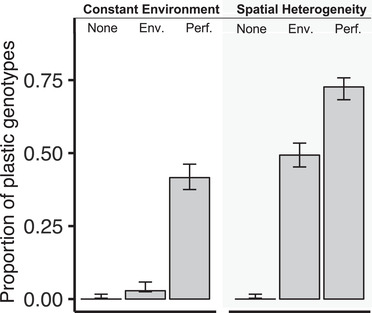
Fraction of genotypes that evolved plasticity in all treatments. The figure excludes simulations where genotypes started plastic. As expected, in the *No Signal* treatment, no genotypes ever evolved plasticity. With signals, a higher fraction of genotypes evolved plasticity in the *Performance Signal* treatment than in the *Environmental Signal* treatment, even when evolved in a constant environment.

With the *environmental signal*, 2.8% of the genotypes starting nonplastic were plastic at the end of the experiment and 18.3% of the genotypes starting plastic were plastic at the end. With the *performance signal*, 41.6% of the genotypes starting nonplastic ended up plastic and 32% of the genotypes starting plastic ended up plastic. Because we are interested in the evolution of plasticity, we report below the fraction of genotypes that evolved plasticity including only simulations where starting genotypes were not plastic. However, including them would not change the conclusion. Excluding the simulations where genotypes started plastic, in the constant environment, we observe that 0%, ∼3%, and ∼42% of the genotypes evolved plasticity in the treatments *No Signal*, *Environmental Signal*, and *Performance Signal*, respectively (Fig. 3). All pairwise differences are significant (Fisher tests: *No Signal* – *Environmental Signal*: *P* < 0.05; *No Signal* – *Performance Signal*: *P =* 2.2 × 10^−16^; *Environmental Signal* – *Performance Signal*: *P =* 6.55 × 10^−16^).

Although the above results are drawn from cases where we test preadaptive plasticity to produce a larger phenotype, we show in Appendix B in the Supporting Information that preadaptive plasticity also evolves in a constant high optimum environment, typically producing genotypes that develop a partially adapted smaller phenotype when grown in the low environment.

Because some of the expectations found in the literature about the effect of plasticity of developmental robustness mention that the implementation of a plastic response requires extra genes that can be a supplemental source of noise, we also report a comparison of the number of genes in different treatments separating the plastic from the nonplastic genotypes (Table S1). Note that with the *Environmental Signal* and *Performance Signal* treatments, genotypes start with three more genes than with *No Signal* treatment (see *Methods*). In an ANCOVA, using the type of signal as covariant, the number of genes is not significantly affected by the presence of environmental heterogeneity (*F* = 5.49, *P* < 0.02) but is significantly higher among plastic genotypes than among nonplastic genotypes (*F* = 44.5, *P* < 4.5 × 10^−11^).

## Discussion

### COST OF PLASTICITY

We hypothesized that plasticity in response to an environmental signal would come at a cost of being less developmentally robust. Because a performance signal involves a negative feedback loop and because such loops have been shown to buffer against developmental noise (Becskei & Serrano, 2000; Bhalla & Iyengar, 1999), we hypothesized that this cost would be attenuated or even reversed when the organisms evolve plasticity via a performance signal.

In agreement with this hypothesis, we observe that plasticity comes at a cost of being developmentally noisy but only when the plastic response is mediated via an environmental signal (Fig. 2). Indeed, a performance signal allowed genotypes to evolve plasticity without the cost of being developmentally noisy. Note, however, that in contrast with our hypothesis, for performance‐based plastic responses, we do not find a significant decrease in developmental stability compared with nonplastic individuals but only the absence of a cost.

Empirical studies investigating the relationship between plasticity and developmental instability have found mixed results (DeWitt, 1998; DeWitt et al., 1998; Lind & Johansson, 2009; Perkins & Jinks, 1973; Scheiner et al., 1991; Tonsor et al., 2013; Van Buskirk & Steiner, 2009; Van Kleunen & Fischer, 2005). We have shown that whether plasticity comes at a cost of developmental instability depends upon the developmental mechanism used to implement plasticity. This phenomenon can in part be the reason for this lack of consensus.

There is also a general lack of empirical evidence of fitness cost of plasticity (Van Kleunen & Fischer, 2005)—whether this cost is mediated by developmental instability or some other phenomenon (e.g., epiphenotypic problem, information acquisition costs, etc.; DeWitt et al., 1998). We want to suggest one reason for the general lack of evidence of fitness cost of plasticity. A genotype can only be called “plastic” with regard to a specific phenotypic trait of interest (see also Wagner, 2005), whereas a fitness cost is a general property of a genotype. If being plastic for a specific trait correlates with not being plastic for another trait, then associating any decrease in developmental robustness to this trait will be misleading. Consider body temperature, for example. To have an environmentally robust (nonplastic) body temperature, traits such as shivering, hair position, vasoconstriction, and other mechanisms of body temperature regulation must respond plastically to temperature variation (Tansey & Johnson, 2015). In this example, searching for a cost of plasticity for body temperature can be misleading as what may be costly are the plastic mechanisms (e.g., shivering, hair position, and vasoconstriction) that regulate temperature. Such situations are likely to be common. Any fitness cost of plasticity is always only meaningful when carefully specifying the trait and when keeping in mind that plasticity for this trait may be positively or negatively correlated with plasticity for another trait.

Given that sensing a performance signal has such an advantage over sensing an environmental signal, it raises a question of why an organism would ever evolve to use an environmental signal rather than performance signal. As an example, consider again the *Daphnia* plastic response to the presence of predators already discussed in the introduction. *Daphnia* cannot determine whether their helmet is long enough to be protected against predators because a failure to produce a long enough helmet would be fatal. In such an example, *Daphnia* has no other choice but to use an environmental signal.

### PREADAPTATION TO HETEROGENEOUS ENVIRONMENTS

Several authors hypothesized that organisms using a performance signal are more likely to respond adaptively to novel environments (Frank, 2011; Hull et al., 2001; Snell‐Rood, 2012; Snell‐Rood et al., 2018). For this reason and because a performance signal can increase developmental robustness (Figs. 1 and S1), we hypothesized that a performance signal mechanism can evolve even in a constant environment and grant the organism an ability to respond adaptively to novel environments. In agreement with this hypothesis, we observed that in the constant environment, plasticity evolved in ∼42% of simulations in the performance signal treatment but in only ∼3% of simulations in the *Environmental Signal* treatment (Fig. 3).

Our hypothesis that a performance signal would help an organism be preadapted for a plastic response in a novel environmental was motivated by the idea that a performance signal would evolve in a constant environment because it would provide a gain in developmental robustness. It is, however, puzzling that plastic genotypes using the performance signal are in fact equally robust than nonplastic genotypes in the same treatment (Fig. S1). We observe that with both a constant environment and with spatial heterogeneity, genotypes using an environmental signal are more developmentally noisy than their nonplastic counterpart. This explains the low propensity of plastic genotypes with a constant environment in the *Environmental signal* treatment. We observe that with both a constant environment and with spatial heterogeneity, genotypes using a performance signal are equally developmentally noisy than their nonplastic counterpart. Using an older version of ENTWINE, Draghi and Whitlock (2015) showed that mutations affecting the mean phenotype also affect the developmental robustness as a correlated effect. We think that with a constant environment in the *Performance signal* treatment, although individual mutations making organisms plastic did not increase developmental robustness on average (data not shown), they caused little to no reduction in developmental robustness and may have been beneficial as a mean to bring the individual's mean phenotype closer to the optimal phenotype. Note that although with the performance signal treatment plastic responses always had a positive reaction norm (adaptive when placed in the heterogeneous environment), this was not the case with the environmental signal treatment (more information on that in Appendix A in the Supporting Information).

Because we frame our work as a proof of concept and also because simulations are computationally very expensive, we did not vary the parameters employed. Our work therefore does not provide reliable estimates of what ought to be observed in nature but only informs about the potential mechanisms at play. For example, our work does not provide an estimate of how much fitness can be gained by using a performance signal versus an environmental signal. Some qualitative results may be affected by varying parameters. For example, if the Gaussian selection kernel were to be set narrower, it could eventually result in more genotypes evolving plasticity.

Historically, the literature on the evolution of plasticity has been focused on the selection pressures acting on the plastic behavior directly and on its associated costs. Indeed, all models for the evolution of adaptive plasticity state that adaptive plasticity can evolve only if populations are exposed to heterogeneous environments and if the different environments select for different phenotypes (e.g., see Bradshaw, 1965; Gomulkiewicz & Kirkpatrick, 1992; Levins, 1968; Lively, 1986; Moran, 1992; Via & Lande, 1985; reviewed in Ghalambor et al., 2007). In our simulations, however, plasticity mediated by a performance signal regularly evolved in a constant environment in contrast to the assumptions of other models in the literature.

Adaptive plasticity has long been suspected of playing a critical role when a species encounters a novel environment (Amarillo‐Suárez & Fox, 2006; Baker, 1974; Baldwin, 1896; Pigliucci & Murren, 2003; Price et al., 2003; Robinson & Dukas, 1999; West‐Eberhard, 2003); reviewed in Ghalambor et al., 2007 and Snell‐Rood et al., 2018) and has been known to be influential in range shifts and invasiveness (Agrawal, 2001; Sultan, 2000; Wennersten & Forsman, 2012; Bock et al., 2018). Our study shows that, when a performance signal is available, a species can evolve to be adaptively plastic for a novel environment that has never been encountered in the species’ evolutionary history. It remains to be estimated how common such processes are in nature.

## DATA ARCHIVING

Data are available at https://doi.org/10.5061/dryad.0gb5mkkzz.

## AUTHOR CONTRIBUTIONS

MCW and JAD conceived the study. All three authors designed the project. JAD and RMD modified the code for ENTWINE. RMD performed the simulations and analyzed the data with feedback from JAD and MCW. RMD wrote the manuscript, which all authors edited.

## FUNDING

The work was funded by NSERC Discovery Grant RGPIN‐2016‐03779 to MCW and by the Swiss National Science Foundation via the fellowship Doc.Mobility P1SKP3_168393 to RMD.

Associate Editor: A. Charmantier

## Supporting information


**Figure S1**: Developmental noise for all treatments at the last generation, separating the plastic (in black) from the non‐plastic genotypes (in grey). The large dot is the mean, and error bars are standard errors.

**Figure S2**: The evolutionary dynamics of developmental robustness over generations. This figure displays the same data as figure 2. Errors bars are standard errors. Simulations are classified as plastic or not plastic based on the genotype considered at the last generation (generation 200k). At each generation displayed, the developmental noise is measured on the most common genotype in the population.

**Table S1**: Comparison of mean and variance in the number of genes among treatments separating the plastic from the non‐plastic genotypes.

**Figure A1**. The reaction norms for evolved genotypes from all treatments.

**Figure B1**. Plasticity patterns for the *Performance Signal* treatment evolved in a constant high environment.
