# Supplementary material for: Plasticity via feedback reduces the cost of developmental instability
Source: Evol Lett. 2020 Nov 19;4(6):570–80. doi: 10.1002/evl3.202 (PMC7719546; doi:10.1002/evl3.202)
Supplement: Supplementary file 1 — Figure S1: Developmental noise for all treatments at the last generation, separating the plastic (in black) from the non‐plastic genotypes (in grey). The large dot is the mean, and error bars are standard errors. [file EVL3-4-570-s001.pdf]

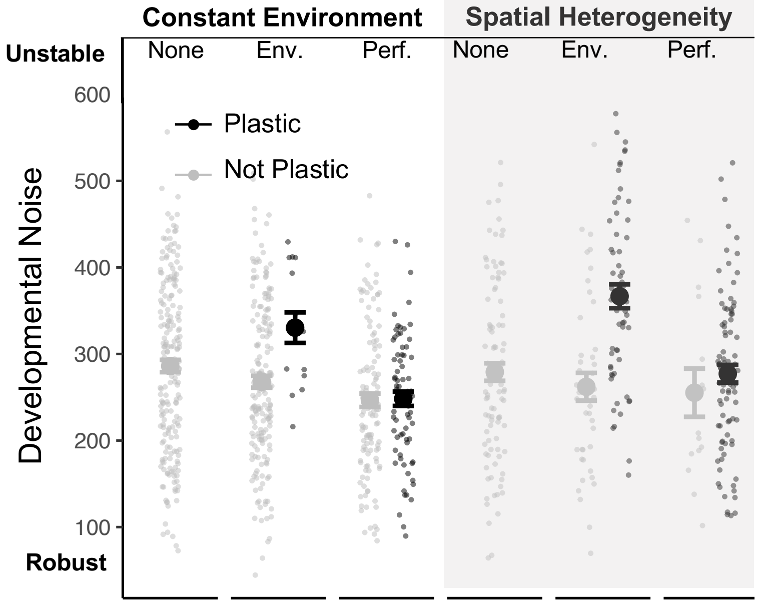
Constant Environment
Spatial Heterogeneity
Unstable
None
Env.
Perf.
None
Env.
Perf.
600
500
400
300
200
100
Robust
Plastic
Not Plastic
Developmental Noise
