# Supplementary figures and images for: Plasticity via feedback reduces the cost of developmental instability
Source: Evol Lett. 2020 Nov 19;4(6):570–80. doi: 10.1002/evl3.202 (PMC7719546; doi:10.1002/evl3.202)

# Spatial Heterogeneity

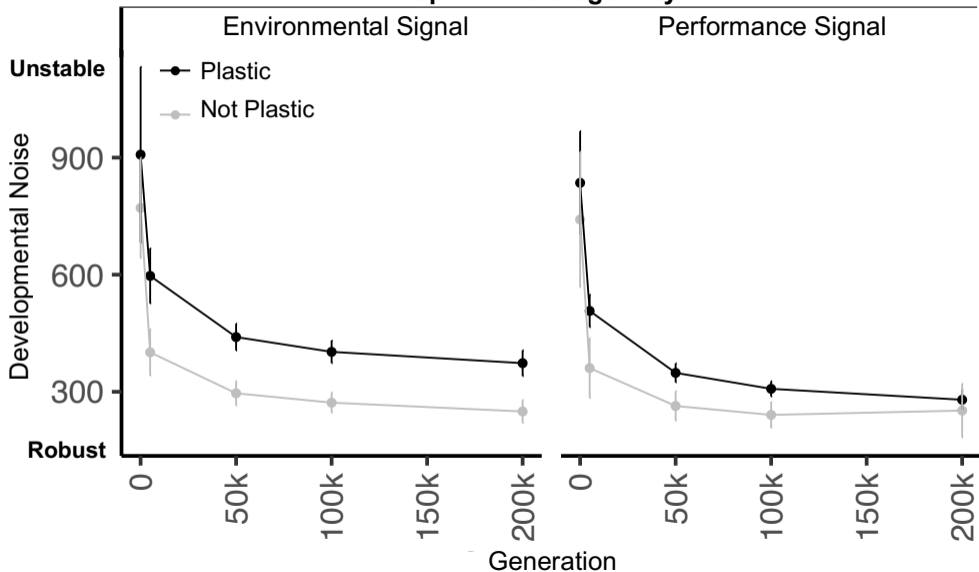

Supplement: Supplementary file 2 — Figure S2: The evolutionary dynamics of developmental robustness over generations. This figure displays the same data as figure 2. Errors bars are standard errors. Simulations are classified as plastic or not plastic based on the genotype considered at the last generation (generation 200k). At each generation displayed, the developmental noise is measured on the most common genotype in the population. [file EVL3-4-570-s002.pdf]
