# Supplementary material for: Plasticity via feedback reduces the cost of developmental instability
Source: Evol Lett. 2020 Nov 19;4(6):570–80. doi: 10.1002/evl3.202 (PMC7719546; doi:10.1002/evl3.202)
Supplement: Supplementary file 3 — Table S1: Comparison of mean and variance in the number of genes among treatments separating the plastic from the non‐plastic genotypes. [file EVL3-4-570-s003.pdf]

Plasticity	Environment	Signal	Initial number of genes	Mean number of genes	Variance number of genes	[min-max]
Plastic	Constant	None	6	-	-	-
Not plastic	Constant	None	6	3.92	3.76	[1-13]
Plastic	Constant	Env. Signal	9	5.93	4.35	[3-10]
Not plastic	Constant	Env. Signal	9	5.05	3.81	[1-13]
Plastic	Constant	Perf. Signal	9	5.17	3.74	[2-11]
Not plastic	Constant	Perf. Signal	9	4.48	2.66	[1-10]
Plastic	Heterogeneous	None	6	-	-	-
Not plastic	Heterogeneous	None	6	3.27	3.27	[1-9]
Plastic	Heterogeneous	Env. Signal	9	5.62	4.42	[2-11]
Not plastic	Heterogeneous	Env. Signal	9	4.07	3.14	[1-8]
Plastic	Heterogeneous	Perf. Signal	9	6.06	4.58	[2-11]
Not plastic	Heterogeneous	Perf. Signal	9	3.66	3.24	[1-7]
