# Supplementary material for: Plasticity via feedback reduces the cost of developmental instability
Source: Evol Lett. 2020 Nov 19;4(6):570–80. doi: 10.1002/evl3.202 (PMC7719546; doi:10.1002/evl3.202)
Supplement: Supplementary file 4 — Figure A1. The reaction norms for evolved genotypes from all treatments. [file EVL3-4-570-s004.docx]

**Appendix A: The directionality of the reaction norm for plastic response involving the environmental signal**

Non-plastic genotypes preferentially evolve to specialize in the low environment (E_o_), in which the starting genotype has been selected to be fit (see *Methods*). We expected that some genotypes in the *Performance Signal* treatment would evolve plasticity in the constant environment as a correlated side effect of evolution for developmental robustness. We also expected that some genotypes in the environmental signal treatment would randomly evolve plasticity. However, we did not expect that among the few genotypes that evolved plasticity in the *Environmental Signal* treatment, the majority would be plastic with a positive reaction norm (as shown below).

In this appendix we propose (and subsequently test) that, with a constant environment, in the *Environmental Signal* treatment, plastic genotypes tend to have a positive reaction norm as an indirect consequence of the growth trajectory in our developmental model. More specifically, in our model, a genotype develops its phenotype starting from zero and its phenotype gene(s) must be activated to build up the phenotype toward the optimal phenotype. The environmental signal can serve as this required activator but only if its cis-regulatory binding site acts as an enhancer and not a repressor. At the start of the simulation, we create a genotype and test its fitness to be between 0.15 and 0.25 in environment 1 (see *Methods -- Starting conditions*). The need of “building up” instead of “building down” a phenotype causes the environmental signal to be preferentially enhancing, rather than repressing, in the selected initial genotypes, causing some genotypes to display a positive (coincidentally pre-adaptive) reaction norm from the beginning of the simulation.

To test the above hypothesis, we evolved populations with a constant environment, in which we give an “*Inverted Environmental Signal*” to individuals (Figure A1, fourth panel). This signal works like the traditional *Environmental Signal* except that it is inputted at a high rate in the low environment (E_o_) and at a low rate in the high environment (E_1_). If our explanation is correct that plastic genotypes (in the constant environment, in the *environmental signal* treatment) are using the environmental signal to start building up their phenotype, then we would expect that when inverting the signal rate of input respective to the environmental value, phenotypes would actually grow smaller when presented into the high environment in a non-adaptive manner. More specifically, we would expect that the fraction of plastic genotypes that have a positive slope without inverting the signals would not be significantly different than the fraction of genotypes that have a negative slope with inverting the signals.

With a constant environment and with the traditional *Environmental Signal*, one genotype is plastic with a negative slope and 15 genotypes are plastic with a positive slope. With the *Inverted Environmental Signal*, three genotypes are plastic with a positive slope and 14 genotypes are plastic with a negative slope. The mean reaction norms are 0.067 and -0.028 for the *Environmental Signal* and the *Inversed Environmental Signal*, respectively. Please note that in the fourth panel, many of the plastic genotypes with a negative reaction norms overlap and cannot be properly distinguished. As expected, the fraction of plastic genotypes with a positive reaction norm in the *Environmental Signal* is not significantly difference from the fraction of plastic genotypes with a negative reaction norms in the *Inverted Environmental Signal* treatment (𝛘^2^; *P*≈0.64). We also observe no significant difference between the mean reaction norm in the *Environmental Signal* and the sign changed mean reaction norm in the *Inverted Environmental Signal* treatment (Kruskal-Wallis, *P*≈0.133).

Figure A1. The reaction norms for evolved genotypes from all treatments. Each line represents a single genotype (from a single independent simulation) and links the average phenotypes in both environments. Reaction norms that are steep enough that we classify them as plastic (see *Methods*) are represented in black; non-plastic genotypes are represented in grey. The horizontal dashed lines represent the optimal phenotypes in each environment. On the right panel are the fitness functions in both environments.
