# Supplementary material for: Plasticity via feedback reduces the cost of developmental instability
Source: Evol Lett. 2020 Nov 19;4(6):570–80. doi: 10.1002/evl3.202 (PMC7719546; doi:10.1002/evl3.202)
Supplement: Supplementary file 5 — Figure B1. Plasticity patterns for the Performance Signal treatment evolved in a constant high environment. [file EVL3-4-570-s005.docx]

**Appendix B: Pre-adaptive plasticity evolving in a constant high optimum environment**

In the main text, we report that many of the genotypes that evolved in a constant environment and in presence of a performance signal can evolve a pre-adaptative plastic response to a novel environment. In the main text, to test for potential pre-adapted plastic responses, we only report simulations where the genotypes evolved in the low environment, then were moved into the high environment. In this appendix, we also evolved genotypes in a constant high environment, and then placed them in the low environment to see if they are also able to evolve pre-adaptive plastic responses.

Because our numerical model is computationally very expensive, we only evolved genotypes in the constant high environment for 5,000 generations. We therefore compared the reaction norms of these simulations with the simulations in the constant low environment, sampled at the same generation.

In the constant low and constant high environments, respectively, 28% and 33% of the most common genotypes in each replicate are plastic (Figure B1). The fraction of plastic genotypes is not significantly different among treatments (Fisher test: *P*≈0.33). Note that all plastic genotypes have an adaptively plastic reaction norm in the constant low environment but not in the constant high. In the constant high environment, 29.5% of genotypes have adaptively plastic reaction norms.

Figure B1. Plasticity patterns for the *Performance Signal* treatment evolved in a constant high environment.
